# Reference Values for Cervical Muscle Strength in Healthy Women Using a Hand-Held Dynamometer and the Association with Age and Anthropometric Variables

**DOI:** 10.3390/healthcare11162278

**Published:** 2023-08-12

**Authors:** Camila Gorla, Taís de Souza Martins, Lidiane Lima Florencio, Carina Ferreira Pinheiro-Araújo, César Fernández-de-las-Peñas, Jaqueline Martins, Débora Bevilaqua-Grossi

**Affiliations:** 1 Department of Health Sciences, Ribeirão Preto Medical School, University of São Paulo, Ribeirão Preto 14049-900, SP, Brazil; camila_gorla@hotmail.com (C.G.); taissomartins@hotmail.com (T.d.S.M.); carinafp@hotmail.com (C.F.P.-A.); jaquelinefisio@usp.br (J.M.); deborabg@fmrp.usp.br (D.B.-G.); 2 Department of Biological and Health Sciences, University of Araraquara, Araraquara 14801-320, SP, Brazil; 3 Department of Physiotherapy, Occupational Therapy, Physical Medicine and Rehabilitation, University of Rey Juan Carlos, 28922 Alcorcón, Spain; cesar.fernandez@urjc.es

**Keywords:** handheld dynamometer, correlation, physical examination

## Abstract

Knowledge of reference values for cervical muscle strength is a key tool for clinicians to use as a clinical reference measure and to establish goals during rehabilitation. The objective was to establish reference values for the maximal strength of cervical muscles in healthy women using a handheld dynamometer and verify the association of cervical muscle strength with age and anthropometric measurements. A hundred women were classified into four groups (*n* = 25) according to age: 18–29 years, 30–39 years, 40–49 years, and 50–60 years. Maximal muscle strength of the cervical spine was measured using a Lafayette^®^ handheld dynamometer for flexion, extension, and bilateral lateral flexion. No differences in cervical muscle strength were observed among the groups (*p* > 0.05). However, the 18–29-year-old group took less time to reach the peak of force for flexion than the 50–60-year-old group. Moderate correlations were observed between cervical flexor strength and weight, body mass index, and neck circumference, and between cervical extensor strength and weight and body mass index (r = 0.43–0.55; *p* < 0.05). Reference values for cervical muscle strength in healthy women were established using a handheld dynamometer, and the association between muscle strength and anthropometric data was moderate.

## 1. Introduction

Neck pain is a common cause of disability, affecting mainly women and adults of active age [1,2]. Neck pain is also the main symptom of cervical dysfunction, which involves several clinical problems with musculoskeletal structures of the cervical spine, such as facet joints and muscles [3]. Cervical dysfunction has been considered one of the most onerous musculoskeletal problems, tremendously impacting individuals’ health and quality of life [4].

Besides pain, different factors can be present in cervical dysfunctions, such as decreased strength in the cervical flexor and extensor muscles [5,6,7,8,9,10]. According to estimations, neck muscles maintain approximately 80% of the mechanical stability of the cervical spine [11], which is essential for holding posture and stabilizing the head. Postural maintenance requires complex and fine integrative coordination involving the central nervous system, neurosensory information, and adequate neuromuscular response. Moreover, postural control and adjustments depend on the stimulus and perturbing external agents [12]. Regarding muscular response and its relationship with cervical posture, it has been reported that strong cervical muscles can even alter the risk of mild traumatic cerebral injury caused by sports and accidents [13]. In contrast, weak cervical muscles are related to the possible development and progression of chronic postural neck pain [14].

Since strength is an important factor in assessing musculoskeletal disorders, knowing regular reference values for different age groups is crucial in determining the level of muscle weakness and in setting goals for the rehabilitation process. Thus, the isometric test of muscle strength is not only an important tool for functional evaluation in rehabilitation and in the estimation of disorder severity, but it can also be useful in objectively assessing the effects of rehabilitation, thus aiding in determining the prognosis for conservative management.

Reference values for cervical muscle strength were already defined for different populations and age groups, but only with fixed measuring devices or adapted fixed dynamometers, with or without stabilization [15,16,17]. However, we only found the use of handheld dynamometers to establish reference values for cervical muscle strength in women of different age groups in a few studies [18,19]. Nevertheless, such studies did not contain details about stabilization and did not assess all the movements in the same sample, conducting separate analyses of the extensor [19] and flexor [18] cervical muscles.

According to the Global Burden of Disease data from 2019, the number of years lived with a disability related to neck pain is higher in women than men [1]. Female sex and a previous history of neck pain are the strongest and most consistent risk factors for chronic neck pain [2]. It is also recognized that some modifiable variables are risk factors for chronic neck pain in women but not men, such as body mass index (BMI) or sleep disorders [20]. Greater prevalence in females is also an epidemiological characteristic of some comorbidities of neck pain, such as temporomandibular disorders or primary headaches (migraine or tension-type headache) [21,22]. Accordingly, reference values for a sample of women would be of interest because it would be the most prevalent sex of patients needing neck muscle rehabilitation in the clinical scenario.

The description of reference values should also consider the association of strength with several individual characteristics. The association of cervical muscle strength with age is still controversial. Some studies did not find any association between strength and age in healthy women [15,16,23], while others suggested that the greater the age, the smaller the expected strength [18,19]. Similarly, the association between anthropometric measurements and cervical muscle strength is controversial. Some studies did not find a correlation between strength, weight, and height in women [16,17], while others observed a moderate or weak correlation of strength with weight, height, and BMI [15,17].

Hence, the objective was to define reference values for cervical muscle strength and the time to reach peak force using the *Lafayette Manual Muscle Testing System* (Lafayette Instrument Company, Lafayette^®^, Lafayette, LA, USA) handheld dynamometer based on distinct age groups. We stabilized the trunk for the following movements performed by healthy women: neck flexion, extension, and lateral flexion. We also assessed the correlation of muscle strength of the cervical spine with age and anthropometric measurements, e.g., weight, height, BMI, and neck length and circumference.

## 2. Materials and Methods

### 2.1. Study Design

This cross-sectional study aimed to compare cervical muscle strength in women of different age groups and verify the association between cervical muscle strength and anthropometric characteristics. We modeled the sample after a pilot study to address the hypothesis test based on the different age groups. We considered α = 0.05 and a power of 80%; thus, a minimal sample of 24 individuals was set for each group. They were divided into four age groups: 18–29 years; 30–39 years; 40–49 years; and 50–60 years. The local ethics committee approved the study protocol, and all participants gave their consent prior to their inclusion in the study (for details, see the “Institutional Review Board Statement” and the “Informed Consent Statement” at the end of the manuscript).

### 2.2. Subjects

We recruited participants from the local and regional community through advertisements, snowball techniques, and among companions of participants in other studies of our research group. Eligibility criteria for the participants’ recruitment from the local community were: women with no history of neck pain, shoulder pain, or headache, aged 18 to 60 years. We also applied a screening questionnaire regarding neck disability related to pain (Neck Disability Index—NDI) [24] and a questionnaire that measured the level of physical activity of the individual (International Physical Activity Questionnaire—IPAQ) [25] for determining sample eligibility according to inclusion and exclusion criteria.

The exclusion criteria were: cervical spine pain during the test, cervical spine pain in the past month that could result in any severity of neck disability (NDI > 4), highly physically active women according to IPAQ, shoulder pain, headache, face or neck surgery or previous trauma, neurological diseases, sensory deficits, systemic diseases that can alter peripheral sensitivity, fibromyalgia, severe heart diseases, and cognitive deficits that hinder communication.

One hundred and three women were recruited; however, when we applied the inclusion and exclusion criteria, three volunteers were excluded for presenting a mild neck disability (NDI score > 4). Thus, 100 women ranging in age from 18 to 60 were divided into four groups according to their age. Each group contained 25 volunteers. All participants in the current study signed the informed consent form before their inclusion.

Demographical and anthropometric data registered were body weight, height, BMI, neck length from C7 to the occipital protuberance with the participant’s chin relaxed towards the chest and the tape measure resting against the natural neck curve, and neck circumference immediately cranial to the thyroid cartilage until C4–C5 vertebral level with the head in a neutral position [15].

### 2.3. Procedures

**Figure 1:**
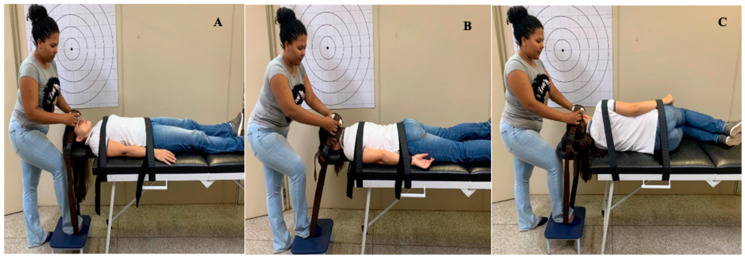
Neck strength measurement proposed in flexion (**A**), extension (**B**), and lateral flexion (**C**) using a handheld dynamometer.

Flexion, extension, and bilateral lateral flexion strength were assessed using a *Lafayette Manual Muscle Testing System* (Lafayette Instrument Company, Lafayette^®^) attached to a non-elastic belt with a velcro^®^ fastener that secured the device in position. Two non-elastic belts with velcro^®^ fasteners were used during all the tests to avoid compensation of other segments. Cervical flexion strength was measured with participants in supine position, with head and neck in neutral position, extended knees, arms alongside the body, and belts fastened over the level of the T3 vertebra and over the anterior superior iliac spine (Figure 1A). Cervical extension strength was measured with volunteers in prone position, head fixed in the face cradle, arms alongside the body, belts stabilizing the pelvic region over the anterior posterior iliac spine, and the fastening over the thorax at T3 level (Figure 1B). Lateral flexion strength was measured with the participants in lateral decubitus position, with an adjustable pillow to maintain the head and neck in neutral position and the belts fastened over the greater trochanter and at T3 level (Figure 1C). All strength measurements were performed by the same examiner (CG), who is a physical therapist with four years of experience, and TSM helped to confirm position and register the data. Both examiners who had trained for the cervical strength measurements had performed approximately 32 h of practice during their preparation for the study. This method has intra-rater (ICC = 0.79–0.90) and inter-rater (ICC = 0.78–0.86) reliability in healthy women, and is considered to have a moderate to excellent level of reliability [26].

Prior to the assessment, the examiner and the participant had a moment of familiarization. Then, the trained examiner taught the movements to the volunteer, who was already in position, and repeated the movement until it was correctly done. Flexion, extension, and bilateral lateral flexion movements were randomized. After being stabilized, the volunteer was instructed to produce maximal strength in each movement direction by verbal standard encouragement. The maximal contraction was sustained for 3 s and repeated 3 times with a 1 min rest between each repetition and a 3 min rest between each change of motion.

### 2.4. Statistical Analysis

All the analysis was done via the statistical software Statistical Package for Social Science for Windows (SPSS) version 20 (SPSS Inc., Chicago, IL, USA). For statistical analyses, we verified the normal distribution of data through the Shapiro–Wilk Test and the equality of variances through the Levene Test. Once the normal distribution was confirmed, we conducted a one-way analysis of variance (ANOVA) for comparisons between age groups. A significance level of 5% was adopted. We described all variables through mean values and confidence intervals of 95% (CI 95%).

We calculated the Spearman Correlation Coefficient to check the correlation between maximal cervical muscle strength and age, and the Pearson Correlation between maximal cervical muscle strength and anthropometric measurements (weight, height, BMI, neck length, and circumference), respectively. The correlation was classified as strong (r ≥ 0.70), moderate (r ≥ 0.40 or r < 0.70), or weak (r < 0.40) [27], with a significance level of 0.05.

## 3. Results

The demographic and anthropometric data of all the groups are presented in Table 1. All the groups showed similar demographic and anthropometric characteristics, except the 50–60-year-old group, which differed from the 18–29-year-old group, presenting greater weight, BMI, and neck circumference.

Table 2 presents data concerning the normalized strength of weight (Kgf/Kg), the time to reach peak force (seconds), and muscle strength minimum and maximum (Kgf) found by each group. According to age, we found no differences between the values of normalized strength in flexion (df = 3/F = 1.495/*p* = 0.221; *p* > 0.05), extension (df = 3/F = 1.068/*p* = 0.366; *p* > 0.05), right lateral flexion (df = 3/F = 1.282/*p* = 0.285; *p* > 0.05), and left lateral flexion (df = 3/F = 2.017/*p* = 0.117; *p* > 0.05). The analysis of time for reaching the peak of muscle strength in each age group revealed differences only in the flexion movement (df = 3/F = 3.150/*p* = 0.029). The group aged 50–60 years showed a longer time to reach their peak force in relation to the youngest group (*p* = 0.018; *p* < 0.05).

Table 3 presents reference values on the maximal isometric cervical muscle strength (Kgf) of the women in each group, assessed with the Lafayette^®^ dynamometer. As for age, we found no differences between the values of muscle strength in flexion (df = 3/F = 2.189/*p* = 0.094; *p* > 0.05), extension (df = 3/F = 0.987/*p* = 0.402; *p* > 0.05), right lateral flexion (df = 3/F = 0.131/*p* = 0.941; *p* > 0.05), and left lateral flexion (df = 3/F = 0.252/*p* = 0.860; *p* > 0.05).

Table 4 presents all the correlation results. We found no significant correlation between cervical muscle strength and age in flexion, extension, or right and left lateral flexion. We noticed a significant moderate correlation between flexion strength and weight (r = 0.55/*p* < 0.001), BMI (r = 0.54/*p* < 0.001), and neck circumference (0.59/*p* < 0.001). A moderately significant correlation was also observed between extension strength with weight *p* < 0.001) and BMI (r_range_ = 0.43–0.46); however, we identified a weak correlation between extension strength with weight and neck circumference (r_range_ = 0.36–0.46). Moreover, we detected other weakly significant correlations between right and left lateral flexion strength with weight, BMI, and neck circumference (r_range_ = 0.22–0.36). We found no significant correlations between muscle strength and height or between muscle strength and neck length.

## 4. Discussion

Our results showed no difference in muscle strength production among groups. Nonetheless, the time for reaching peak force in flexion was different for both the youngest and the eldest groups. The 50–60-year-old group took a longer time to reach the peak than the youngest women (18–29 years). We verified that there is no significant correlation between muscle strength and age. Also, we verified a moderate correlation between flexion strength and weight, BMI, and neck circumference, and between extension strength and weight and BMI.

Our data corroborate the literature [15,16,17,23] since we observed no differences in cervical muscle strength between age groups or association between age and cervical muscle strength in healthy women. Thus, specifically when considering cervical strength, the increase in age does not correlate linearly and prominently with strength. The studies that found correlations between age and strength assessed different age groups, such as strength increases and aging in young athletes [28] and strength decreases in women over 60 years [19,29]. However, Phillips et al. [18] observed a strength decrease in the 50–59-year-old group but solely evaluated cervical flexors. Previous results demonstrated that cervical muscle strength is maintained for at least 70 years [16,23]. Although we have found no difference in muscle strength among age groups, the youngest group reached the peak of flexion strength in less time than the oldest group, displaying a better performance with a quicker and more agile response in a protocol of 3 s of contraction. Nevertheless, we cannot affirm that these differences would be maintained if participants had to sustain muscular contraction for a longer interval. However, this time-related response may influence neuromuscular response to external perturbations to adapt and maintain cervical posture, as postural control adaptations vary depending on the stimulus [12].

Our results for the association of cervical muscle strength with anthropometric measurements concur with those of Salo et al. [17] and Catenaccio et al. [15], which also observed a weak correlation of some anthropometric measurements with cervical muscle strength. Thus, there seems to be no significant association regarding lateral flexion movements in clinic practice. On the other hand, Garces et al. [29] demonstrated a strong correlation of height with flexion and extension in women differently from the present study, which found no association of cervical muscle strength with such an anthropometric measurement. Although Chiu et al. [23] did not find a correlation between weight or height and strength, our data showed a moderate correlation between flexion and weight, BMI, and neck circumference and a moderate correlation between extension and weight and BMI in women. In addition, our data suggest that the greater the muscle mass, BMI, and neck circumference, the greater the cervical muscle flexion strength, except in the 50–60-year-old group. We can observe the same regarding cervical extension, weight, and BMI. Therefore, there seems to be a relationship between cervical muscle strength and anthropometric measurements, but not linearly.

As to our knowledge, our study is pioneering in presenting reference values on cervical muscle strength and the time for reaching the peak force in healthy women utilizing a handheld dynamometer (Lafayette^®^), with detailed stabilizations and in movements of flexion, extension, and right and left lateral flexion. In this manner, according to the reference value for each group, setting better goals for the process of rehabilitation of the cervical region becomes possible since strength is a useful indicator of cervical function [30]. Moreover, reference values can be an alternate reference for determining the proportional value of progressive load applied in protocols for cervical muscle strengthening. In this manner, the basic principles of a strengthening protocol can be adapted without calculating the load of a maximal repetition. In clinical practice, such calculations for cervical muscle strength are not functional.

As for reference data on cervical muscle strength using handheld dynamometers available in the literature, Staudte and Duhr [19] reported data for cervical extension utilizing the same dynamometer as this study, and Phillips et al. [18] reported data for flexion with a Penny and Giles^®^ dynamometer. Table 5 presents reference values reported by these two studies, and our results, which were transformed into Newton (N) to facilitate a qualitative comparison with the present results. Our study’s reference values for cervical flexion strength are smaller than those described by Philips et al. [18]. Methodological differences might have contributed to these findings, such as using another dynamometer and only one test repetition. However, the reference values reported by Staudte and Duhr [19] regarding extension using the same dynamometer cannot be directly compared with our data, fundamentally because the age range of their groups is completely different from ours. Additionally, each group had a varied sample size, thus being less representative of more advanced age groups. Also, none of the two studies provided details on stabilization, which may have affected the registered strength. Considering lateral flexion values, it was impossible to compare them because the present study is the first to define them with a handheld dynamometer.

Thus, we emphasize that our study provides reference values on the strength of cervical muscles in various directions utilizing the same handheld dynamometer. Moreover, the applied method used two non-elastic belts for stabilization to decrease the compensation of trunk muscles in the strength registered by cervical muscles, thus attenuating the main criticism of using handheld dynamometers [31]. Thereby, we can provide practitioners with reference values obtained through reliable methods that may be useful in the rehabilitation process [26], in estimating the deficit generated by a head or neck dysfunction, and even in preventing and detecting occupational problems related to cervical dysfunction. Since it is a portable device that is easy to use in the clinical environment, it could also be utilized in ergonomic assessments in loco for companies.

The handheld dynamometer is not always the best option to measure strength in all scenarios. In clinical and experimental settings, strength can be measured by the maximal force produced during an isometric contraction, the maximal load that can be lifted once or during ten repetitions, or by the peak torque produced by an isokinetic contraction [31]. Some clinicians also use manual muscle testing to estimate muscle strength, although it is a criticized method [32]. It will depend on the functionality of the individual, the objective of the assessment, the type of contraction, the segment of interest, and the availability of tools. For example, for grip strength, you can use a specific hand dynamometer; for pinch strength, it is recommended to use a spring gauge [33,34]. The gold standard for lower limb strength assessment is the isokinetic dynamometer [35]. Considering the cervical segment, the strength measures available would be fixed-framed dynamometers, handheld dynamometers, isokinetic dynamometers, or manual muscle testing [31]. According to Strimpakos et al. [31], they all have limitations due to subjectivity, low reliability, or a lack of appropriate stabilization. As mentioned above, the method used to measure the strength of neck muscles in our study attempts to provide more trunk stability and presents adequate reliability [26].

The limitations of this study are related to the profile of the sample. Since only women were assessed, the results of association and correlation cannot be generalized to men. It is justified by previous studies that have identified differences between women and men when dealing with cervical strength [15,18,19,23,29]. Moreover, once excluded from this sample, our data cannot be extrapolated to highly physically active women. Another limitation that should be considered is the absence of a gold standard for an ideal sample size for normative data. Herein, we calculated the sample to compare age groups, so the sample size is adequate to address this specific question. We named our data as reference values instead of normative data, considering this limitation. Accordingly, the reference values can be interpreted by the mean of each age subgroup and by its 95% CI. The narrow range of the 95% CI presented in the reference values of Table 3 may give more precision in terms of extrapolation.

## 5. Conclusions

Cervical muscle strength may not differ among age groups; however, women aged 50–60 years take longer to produce peak force than women aged between 18–29 years.

In women with no complaints of pain or disability in the cervical area, and greater weight, BMI, and neck circumference, greater strength is expected, especially in cervical flexion and extension.

## Figures and Tables

**Table 1:** Mean and standard deviation of demographic and anthropometric data sample.

Groups (*n* = 25)	Age (Years)	Height (m)	Weight (Kg)	BMI (Kg/m²)	Neck Length (cm)	Neck Circumference (cm)
18–29 years	22.0 (3.17)	1.63 (0.07)	64.28 (10.71)	24.16 (3.91)	14.76 (1.74)	33.34 (1.92)
30–39 years	33.5 (3.1)	1.65 (0.06)	69.66 (13.64)	25.57 (4.92)	15.46 (1.45)	33.98 (2.32)
40–49 years	43.4 (3.59)	1.62 (0.06)	70.44 (11.35)	26.94 (4.67)	14.48 (1.34)	35.14 (3.02)
50–60 years	55.4 (3.83)	1.63 (0.06)	74.6 (14.53) *	28.20 (5.01) *	14.66 (0.94)	36.16 (3.19) *

BMI: body mass index; m: meter; Kg: kilogram; cm: centimeter; * *p* < 0,05 for the Bonferroni method post-hoc in comparison to the 18–29-year-old group.

**Table 2:** Data about strength normalized by weight, minimal and maximal strength, and the time to reach the peak of strength.

		Normalized Strength (kgf/Kg)	Minimal (kgf)	Maximum (kgf)	Time to Reach the Peak (s)
18–29 years (*n* = 25)	Flexion.	0.08 (0.07–0.08)	2.7	6.9	1.99 (1.71–2.26)
	Extension	0.15 (0.13–0.16)	5.4	13.1	2.49 (2.27–2.70)
	RLF	0.11 (0.10–0.12)	3.8	12.6	2.24 (2.01–2.47)
	LLF	0.11 (0.10–0.13)	3.6	11.2	2.18 (1.96–2.40)
30–39 years (*n* = 25)	Flexion	0.08 (0.07–0.09)	3.0	14.4	2.24 (2.00–2.48)
	Extension	0.14 (0.13–0.15)	5.8	13.5	2.53 (2.34–2.72)
	RLF	0.10 (0.09–0.11)	4.0	10.7	2.63 (2.19–3.00)
	LLF	0.10 (0.10–1.11)	3.6	9.4	2.41 (2.25–2.70)
40–49 years (*n* = 25)	Flexion.	0.09 (0.08–0.10)	2.2	13.9	2.29 (2.14–2.44)
	Extension	0.15 (0.14–0.16)	8.2	13.2	2.39 (2.21–2.57)
	RLF	0.10 (0.09–0.11)	2.8	13.4	2.24 (2.06–2.42)
	LLF	0.11 (0.10–0.12)	3.6	14.2	2.34 (2.17–2.51)
50–60 years (*n* = 25)	Flexion	0.08 (0.07–0.09)	2.9	10.9	2.46 (2.29–2.63) *
	Extension	0.13 (0.13–0.14)	7.1	12.6	2.51 (2.40–2.62)
	RLF	0.10 (0.09–0.10)	5.0	10.8	2.31 (2.11–2.50)
	LLF	0.09 (0.00–0.10)	4.8	11.4	2.34 (2.17–2.51)

All values are mean (95% confidence interval); RLF: right lateral flexion; LLF: left lateral flexion; Kgf: kilogram-force; Kg: kilogram; * *p* < 0.05 for Bonferroni method post-hoc in comparison to the 18–29-year-old group, concerning the time to reach their peak of muscle strength.

**Table 3:** Reference values for women’s neck maximal muscle strength using handheld dynamometer (Lafayette^®^).

Groups (*n* = 25)	Flexion (kgf)	Extension (kgf)	RLF (kgf)	LLF (kgf)
18–29 years	4.94 (4.45–5.42)	9.30 (8.53–10.07)	6.99 (6.21–7.76)	7.19 (6.44–7.93)
30–39 years	5.53 (4.65–6.41)	9.49 (8.71–10.27)	7.02 (6.40–7.64)	7.16 (6.60–7.72)
40–49 years	6.33 (5.41–7.25)	10.05 (9.56–10.55)	7.24 (6.40–8.08)	7.38 (6.53–8.24)
50–60 years	5.77 (5.06–6.47)	9.85 (9.25–10.44)	6.95 (6.36–7.53)	6.94 (6.32–7.57)
ANOVA *p* value	0.094	0.402	0.941	0.860

All values are mean (95% confidence interval); RLF: right lateral flexion; LLF: left lateral flexion; kgf (kilogram-force).

**Table 4:** Correlation between neck muscle strength, age, and anthropometric data determined by the Spearman correlation coefficient (*r*).

		Age (Years)	Height (m)	Weight (Kg)	BMI (Kg/m²)	Neck Length (cm)	Neck Circumference (cm)
Flexion.	*r*	0.14	0.06	0.55 *	0.54 *	0.11	0.59 *
	*p* value	0.170	0.568	<0.001	<0.001	0.274	<0.001
Extension	*r*	0.11	0.07	0.46 *	0.43 *	0.09	0.36 *
	*p* value	0.283	0.494	<0.001	<0.001	0.394	<0.001
RLF	*r*	0.01	0.19	0.28 *	0.22 *	0.15	0.31 *
	*p* value	0.946	0.057	0.004	0.029	0.139	0.002
LLF	*r*	−0.11	0.13	0.34 *	0.29 *	0.19	0.36 *
	*p* value	0.282	0.213	0.001	0.003	0.057	<0.001

* Significant correlation given *p* value < 0.05.

**Table 5:** The comparison between reference values on cervical strength in flexion and extension using a handheld dynamometer available in the literature.

Flexion				Extension			
Phillips et al. [18] (*n* = 98)	Strength (N)	Current Study (*n* = 100)	Strength (N) ^a^	Staudte and Duhr [19] (*n* = 163)	Strength (N)	Current Study (*n* = 100)	Strength (N) ^a^
20–29 years (*n* = 20)	82 (13)	18–29 years (*n* = 25)	48(12)	14–24 years (*n* = 75)	68.8 (24)	18–29 years (*n* = 25)	91.2 (19.3)
30–39 years (*n* = 18)	86 (13)	30–39 years (*n* = 25)	54 (22)	25–34 years (*n* = 27)	76.5 (25.6)	30–39 years (*n* = 25)	93.0 (19.5)
40–49 years (*n* = 20)	78 (14)	40–49 years (*n* = 25)	62(23)	35–44 years (*n* = 14)	70.2 (19.4)	40–49 years (*n* = 25)	98.5 (12.4)
50–59 years (*n* = 20)	72 (15)	50–60 years (*n* = 25)	57 (18)	45–54 years (*n* = 8)	71.9 (17.1)	50–60 years (*n* = 25)	96.6 (14.0)
60–69 years (*n* = 20)	66 (13)	--	--	55–64 years (*n* = 6)	56.1 (19.8)	--	--
				65–74 years (*n* = 10)	57.2 (16.9)	--	--
				>74 years (*n* = 3)	44.6 (24.7)	--	--

All values are mean (standard deviation); N: newton. ^a^ Data were converted in Newtons to allow indirect comparison to literature.

## Data Availability

The data presented in this study are available on request from the corresponding author. The data are not publicly available due to ethical issues.
